# Hormaomycins B and C: New Antibiotic Cyclic Depsipeptides from a Marine Mudflat-Derived *Streptomyces* sp.

**DOI:** 10.3390/md13085187

**Published:** 2015-08-14

**Authors:** Munhyung Bae, Beomkoo Chung, Ki-Bong Oh, Jongheon Shin, Dong-Chan Oh

**Affiliations:** 1 Natural Products Research Institute, College of Pharmacy, Seoul National University, Seoul 151-742, Korea; E-Mails: baemoon89@snu.ac.kr (M.B.); shinj@snu.ac.kr (J.S.); 2 Department of Agricultural Biotechnology, College of Agriculture and Life Science, Seoul National University, Seoul 151-921, Korea; E-Mails: beomkoo01@snu.ac.kr (B.C.); ohkibong@snu.ac.kr (K.-B.O.)

**Keywords:** marine actinomycete, secondary metabolite, hormaomycin, peptide, antibiotic

## Abstract

Alterations in microbial culture conditions may trigger the production of diverse bioactive secondary metabolites. While applying various culture conditions and monitoring secondary metabolite profiles using LC/MS, hormaomycins B and C (**1** and **2**) were discovered from a marine mudflat-derived actinomycete, *Streptomyces* sp., collected in Mohang, Korea. The planar structures of the hormaomycins, which bear structurally-unique units, such as 4-(*Z*)-propenylproline, 3-(2-nitrocyclopropyl)alanine, 5-chloro-1-hydroxypyrrol-2-carboxylic acid and β-methylphenylalanine, were established as the first natural analogues belonging to the hormaomycin peptide class. The absolute configurations of **1** and **2** were deduced by comparing their CD spectra with that of hormaomycin. These hormaomycins exhibited significant inhibitory effects against various pathogenic Gram-positive and Gram-negative bacteria.

## 1. Introduction

Microbial secondary metabolites have been regarded as a major source of antibiotics over the past few decades [1,2]. However, the decline in the efficiency of discovering antibiotics from terrestrial microbes has increased natural products chemists’ interest in investigating the chemistry of marine microorganisms [3,4], particularly as antibiotic resistance emerges as a significant threat to human health [5]. Although only a small portion of marine microbes have been chemically studied because of limited accessibility and technical problems, the discovery of promising drug leads, including an anticancer drug candidate, salinosporamide A [6], and an antibiotic drug candidate, thiocoraline [7], from marine actinomycetes, has revealed that marine actinomycetes are particularly prolific in their production of structurally- and biologically-unique secondary metabolites with pharmaceutical potential.

**Figure 1:**
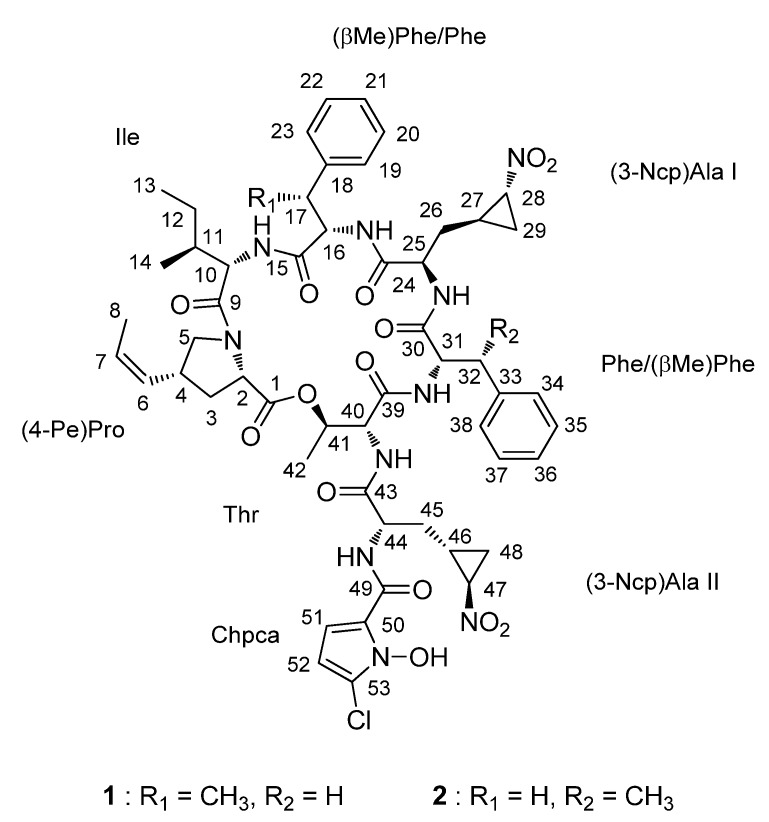
The structures of hormaomycins B and C (**1** and **2**).

As part of our efforts to search for new bioactive compounds, actinomycete strains were selectively isolated from uncommon marine environments, such as salterns, the Arctic sea and deep-sea sediments. The application of a chemical analysis-based discovery strategy using LC/MS chemical profiles allowed us to discover unique bioactive secondary metabolites in various structural class [8,9,10]. Based on genomic analyses of actinomycetes, the biosynthetic capacity of an actinomycete strain is considerably greater than the number of biosynthetic pathways turned on under a particular culture condition [11]. As the “OSMAC (One Strain Many Compounds)” strategy, which was developed to maximize microbial chemical diversity, suggests, altering the culture conditions of a microbial strain may trigger silent biosynthetic pathways [12]. In our previous report, the actinomycete strain SNM55, which was isolated from the intertidal zone mudflat in Mohang, Korea, was reported to produce the structurally-novel pseudodimeric peptides mohangamides, consisting of 14 amino acids and two unusual acyl chains [13]. The secondary metabolites of the strain SNM55 were further examined by changing the culture medium, time and other conditions. The chemical components of the SNM55 culture extracts were monitored using LC/MS almost every day. During a prolonged cultivation of the strain for 10 days, which is two-times longer than the cultivation time required for mohangamide production, another series of compounds, each bearing a chlorine atom, was detected by LC/MS. Scaling up the SNM55 culture and performing further chromatographic isolation yielded pure compounds. Subsequent spectroscopic analysis revealed that these compounds are new cyclic depsipeptides belonging to the hormaomycin class. Hormaomycin is a highly-modified antibiotic peptide that was originally identified from *Streptomyces griseoflavus* [14]. Despite the early report of its structure in approximately 1990, no natural congeners have been reported for hormaomycin. Here, we report hormaomycins B and C (**1** and **2**) (Figure 1), which were produced by altering the culture conditions of the marine *Streptomyces* strain SNM55, as the first new natural hormaomycin analogues and their antimicrobial activity against various pathogenic bacteria.

## 2. Results and Discussion

### 2.1. Structural Elucidation

Hormaomycin B (**1**) was isolated as a white powder with the molecular formula C_54_H_67_ClN_10_O_14_ on the basis of HRFABMS (obsd. [M + H]^+^ at *m*/*z* 1115.4611, calcd. for 1115.4605) coupled with ^1^H and ^13^C NMR spectroscopy (Table 1). The ^1^H spectrum of **1** in CDCl_3_ presented six amide protons at δ_H_ 8.98, 8.16, 7.31, 6.85, 6.79 and 6.26, indicating its peptide-derived nature. Consistently, the ^13^C NMR of **1** also showed seven amide/ester carbons at δ_C_ 172.2, 171.8, 171.5, 171.1, 169.3, 168.8 and 167.9. The analysis of ^1^H, ^13^C and HSQC NMR spectra clarified the peptide-derived features of hormaomycin B. As a peptide-derived molecule, hormaomycin B exhibited seven methine signals composing α-positions of amino acids (δ_H_/δ_C_ 5.14/51.2, 3.52/52.2, 4.64/54.5, 4.55/58.4, 4.48/56.4, 4.33/60.8 and 4.26/61.7). Seven amide/ester carbons and seven α-position methines enabled us to deduce that hormaomycin B (**1**) bears seven or more amino acid units. Further analysis of one-bond correlations identified NMR signals that do not belong to ordinary amino acids units. In particular, two olefinic methines (δ_H_/δ_C_ 5.64/128.8 and 5.26/127.8) and two methines (δ_H_/δ_C_ 0.28/20.2 and −0.66/17.7) and one methylene (δ_H_/δ_C_ 0.56 and −0.26/33.4) in the distinctive upfield suggested the existence of unusual units in the molecules. Additionally, two aromatic methines (δ_H_/δ_C_ 6.68/109.8 and 6.08/103.5) with 4.5 Hz ^1^H-^1^H coupling indicated the existence of a five-membered aromatic ring, which does not correspond to any proteinogenic amino acid. Based on the overall analysis of the ^1^H, ^13^C NMR and HSQC spectra of **1**, along with the existence of a chlorine atom in the molecular formula, hormaomycin B was expected to be a peptide-derived compound bearing several highly-modified amino acid residues.

Further analysis of HSQC spectral data revealed all of the one-bond ^1^H-^13^C correlations in **1**. Subsequent interpretation of the COSY, TOCSY and HMBC NMR spectra established eight partial structures dissected by amide bonds. These include a 4-(*Z*)-propenylproline ((4-Pe)Pro), a β-methylphenylalanine ((β-Me)Phe), two 3-(2-nitrocyclopropyl)alanines ((3-Ncp)Ala) and a 5-chloro-1-hydroxypyrrol-2-carboxylic acid (Chpca), as well as three common amino acids: an isoleucine (Ile), a phenylalanine (Phe) and a threonine (Thr).

**Figure 2:**
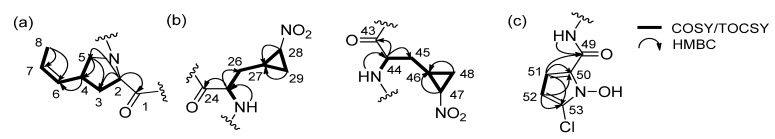
Determination of the unusual partial structures of **1**. (**a**) 4-*(Z)*-propenylproline (**b**) 3-(2-nitrocyclopropyl)alanine I and II and (**c**) 5-chloro-1-hydroxypyrrol-2-carboxylic acid.

Specifically, the COSY NMR spectrum exhibited correlations from H_2_-3 (δ_H_ 2.37, 1.80) to H-2 (δ_H_ 4.26) and H-4 (δ_H_ 3.27), showing the connectivity from C-2 (δ_C_ 61.7) to C-4 (δ_C_ 36.9) through C-3 (δ_C_ 34.9). Further COSY correlations of H-4 to H_2_-5 (δ_H_ 3.98, 3.31) and H-6 (δ_H_ 5.26) revealed that C-4 was located immediately adjacent to C-5 (δ_C_ 53.0) and C-6 (δ_C_ 127.8). The ^1^H-^1^H couplings from H-7 (δ_H_ 5.64) to H-6 and H-8 (δ_H_ 1.68) and HMBC correlations from H-8 to two olefinic carbons C-6 and C-7 (δ_C_ 128.8) established the propenyl substructure connected to C-4. The ^1^H-^1^H coupling constant (*J* = 9.5 Hz) between H-6 and H-7 determined the Z configuration of the C-6 double bond. The ^13^C NMR chemical shift of C-5 (δ_C_ 53.0) indicated that this carbon is bound to a nitrogen atom. Because there was no detected NH signal in this unit, a proline-type moiety was expected. The HMBC correlation from theα-proton (H-2; δ_H_ 4.26) to C-5 supported the hypothesis that H-2 and H-5 share one nitrogen to form a pyrrolidine. Furthermore, H-2 displayed an HMBC correlation to the C-1 carbonyl carbon (δ_C_ 171.8), completing a 4-(*Z*)-propenylproline ((4-Pe)Pro) unit (Figure 2a). This unit was further confirmed by TOCSY correlations from H-2 to H-8.

The next unusual unit was composed of distinctively-shielded protons of H_2_-26 (δ_H_ 0.56, −0.26), H-27 (δ_H_ 0.28) and H_2_-29 (δ_H_ 1.02, −0.66), along with 25-NH (δ_H_ 6.26), H-25 (δ_H_ 3.52) and H-28 (δ_H_ 2.92), based on COSY and TOCSY NMR spectra. In particular, strong COSY correlations among H-27, H-28 and H_2_-29 and their low chemical shifts revealed that this amino unit possesses a cyclopropane. Additionally, the ^13^C chemical shift (δ_C_ 58.7) of the C-28 methine in the cyclopropane indicated that this carbon is directly connected to a heteroatom. The IR spectrum exhibited a strong absorption band at 1543 cm^−1^, which corresponds to a nitro functional group. Therefore, C-28 was determined to bind to a nitro group. These assignments eventually elucidated a 3-(2-nitrocyclopropyl)alanine ((3-Ncp)Ala) unit (Figure 2b). Interestingly, the second (3-Ncp)Ala unit was also identified based on COSY and TOCSY correlations, even though the ^1^H chemical shifts of the second unit differ significantly from those of the first (3-Ncp)Ala residue (Figure 2b, Table 1).

Hormaomycin B (**1**) possesses eighteen sp^2^ carbons. Twelve of these were assigned in the two benzene rings in a phenylalanine and a β-methylphenylalanine, and two olefinic carbons were used in (4-Pe)Pro. Then, four sp^2^ carbons, including two methines, C-51 (δ_C_ 109.8) and C-52 (δ_C_ 103.5), and two quaternary carbons, C-50 (δ_C_ 119.8) and C-53 (δ_C_ 121.5), remained unassigned. These methine protons, H-51 (δ_H_ 6.68) and H-52 (δ_H_ 6.08), were coupled to each other (4.5 Hz). This coupling constant strongly suggested that these atoms are part of a five-membered aromatic ring. Furthermore, HMBC correlations from H-51 to C-50 and C-53 and from H-52 to C-50 and C-53 established the C-50-C-51-C-52-C-53 connectivity. The last position of the five-membered aromatic ring could be a nitrogen atom rather than an oxygen or sulfur atom based on the ^13^C chemical shifts of the four sp^2^ carbons, indicating a pyrrole ring [15]. This pyrrole ring is connected to C-49 (δ_C_ 159.7) at C-50 according to the HMBC correlation from H-51 to C-49. This downfield carbon at δ_C_ 159.7 was assigned as an amide carbonyl carbon based on the heteronuclear coupling from the amide proton 44-NH (δ_H_ 8.16) to this carbon. The remarkable shielding of C-49 (δ_C_ 159.7) in the ^13^C NMR spectrum compared to the chemical shifts of the other seven amide carbons (δ_C_ 172.2, 171.8, 171.5, 171.1, 169.3, 168.8 and 167.9) might be attributed to the conjugation effect of the pyrrole ring. Detailed examination of the mass and NMR data suggested that the quaternary carbon C-53 and the nitrogen atom in the pyrrole ring should be substituted with heteroatoms. Finally, partial structures dissected by amide bonds were established, and one chlorine atom and one hydroxy group in the molecular formula were not assigned. Considering the chemical shift of C-53 enabled a chlorine atom to be located at C-53. Subsequently, the hydroxy group was assigned to be bound to the nitrogen atom as an oxime functional group. Therefore, 5-chloro-1-hydroxypyrrol-2-carboxylic acid (Chpca) was eventually assembled (Figure 2c).

**Figure 3:**
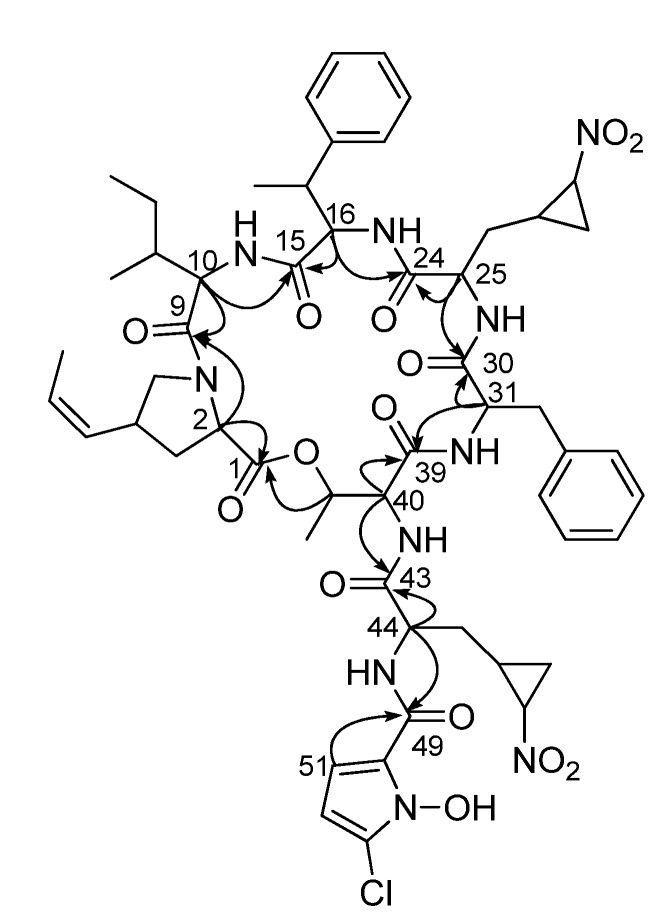
Key HMBC correlations of hormaomycin B (**1**).

The sequence of eight partial structures ((4-Pe)Pro, Ile, Phe, (βMe)Phe, (3-Ncp)Ala I and II, Thr and Chpca) was determined on the basis of HMBC correlations. The long-range ^1^H-^13^C coupling from the α-proton of (4-Pe)Pro (H-2; δ_H_ 4.26) to the carbonyl carbon of Ile (C-9; δ_C_ 171.5) connected (4-Pe)Pro to Ile. The HMBC correlations from 10-NH (δ_H_ 7.31) and H-16 (δ_H_ 4.33) to C-15 (δ_C_ 167.9) clarified the attachment of (βMe)Phe to Ile. (3-Ncp)Ala I was directly connected to (βMe)Phe based on the HMBC correlations from 16-NH (δ_H_ 6.85) and H-25 (δ_H_ 3.52) to C-24 (δ_C_ 169.3), which belonged to (3-Ncp)Ala I. Phe was positioned next to (3-Ncp)Ala I by ^1^H-^13^C long-range coupling from NH-25 (δ_H_ 6.26) and H-31 (δ_H_ 4.48) to C-30 (δ_C_ 168.8). The HMBC correlations from 31-NH (δ_H_ 6.79) and H-40 (δ_H_ 4.55) to C-39 (δ_C_ 171.1) revealed the sequence from Phe to Thr. In addition, the NH proton (δ_H_ 8.98) of Thr and the α-proton (δ_H_ 5.14) of (3-Ncp)Ala II displayed HMBC signals linking them to the carbonyl carbon C-43 of (3-Ncp)Ala II, clarifying the connection between Thr and (3-Ncp)Ala II. Chpca was directly connected to (3-Ncp)Ala II on the basis of the HMBC correlations from 44-NH (δ_H_ 8.16) of (3-Ncp)Ala II and H-51 (δ_H_ 6.68) of Chpca to the carbonyl carbon C-49 (δ_C_ 159.7) belonging to Chpca. Finally the long-range HMBC correlation from H-41 (δ_H_ 5.40) to the carbonyl carbon at 171.8 ppm (C-1) closed a macrocyclic lactone ring, indicating the planar structure of hormaomycin B (**1**) in a cyclic depsipeptide (Figure 3).

Hormaomycin C **(2**) was isolated as a white powder, and the molecular formula was deduced as C_54_H_67_ClN_10_O_14_ by analyzing HRFABMS (obsd. [M + Na]^+^ at *m*/*z* 1137.4432, calcd. [M + Na]^+^ 1137.4424) and ^1^H and ^13^C NMR data (Table 1). The molecular formula of **2** was identical to that of hormaomycin (**1**), and it was predicted that hormaomycin C (**2**) would be very similar to **1**. Careful comparison of 1D and 2D NMR data revealed that all eight partial structures identified in hormaomycin B also exist in **2**. However, the extensive analysis of the HMBC NMR spectra showed that the sequence of the units was different. Specifically, the methyl group (17-Me; δ_H_ 1.29) of (βMe)Phe and the methylene signal (H_2_-32; δ_H_ 2.79, 3.38) of Phe were replaced with a methylene (H_2_-17; δ_H_ 2.91, 2.97) and a methyl group (32-Me; δ_H_ 1.40) in **2**, indicating the switched positions of (βMe)Phe and Phe between these compounds. The order of the other units was assigned based on further HMBC spectroscopic analysis, completing the planar structure of hormaomycin C (**2**).

**Figure 4:**
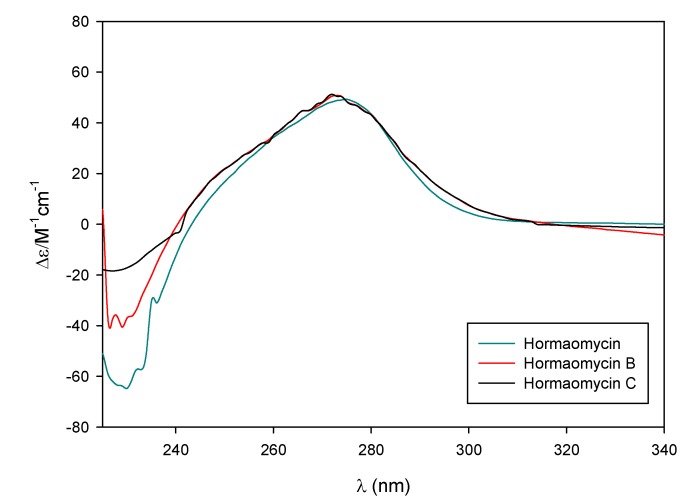
The CD spectra of the hormaomycins in MeOH.

During cultivation of the strain SNM55, hormaomycin, which was previously discovered from *Streptomyces griseoflavus* [14], was identified along with these new compounds. Hormaomycin is a cyclic depsipeptide that bears almost exclusively uncommon amino acids, such as (4-Pe)Pro and (3-Ncp)Ala, as well as Chpca. Because of its structural novelty and remarkable biological activity, chemical synthesis [16] and biosynthetic modifications [17] have been studied in recent decades. However, although artificial analogues have been reported during the total synthesis and biosynthetic engineering of hormaomycin [16,17], no natural analogues have been discovered. To our best knowledge, hormaomycins B and C are the first natural analogues of hormaomycin that have been obtained without chemical modification or gene cluster manipulation. Hormaomycin possesses two (βMe)Phe units, whereas both hormaomycins B and C bear one (βMe)Phe and one Phe. Therefore, hormaomycin was utilized to determine the absolute configurations of hormaomycins B and C (**1** and **2**). The ^1^H and ^13^C NMR data and CD spectrum of hormaomycin were carefully compared to those of **1** and **2**. Based on the close similarity between the NMR data and CD spectra (Figure 4), as well as their common biosynthetic origin, the absolute configurations of hormaomycins B and C were determined to be identical to that of hormaomycin, which was determined by total synthesis [16].

**Table 1:** ^1^H (600 MHz) and ^13^C NMR (150MHz) data for hormaomycins B and C (**1** and **2**) in CDCl_3_.

Unit	C/H	1			2		
		δ_H_, mult (*J* in Hz)	δ_C_	Type	δ_H_, mult (*J* in Hz)	δ_C_	Type
(4-Pe)Pro	1	–	171.8	C	–	171.6	C
	2	4.26, 1H, m	61.7	CH	4.26, 1H, dd (11.0, 6.5)	61.8	CH
	3	2.37, 1H, m	34.9	CH_2_	2.36, 1H, m	35.8	CH_2_
		1.80, 1H, m			1.81, 1H, m		
	4	3.27, 1H, m	36.9	CH	3.25, 1H, m	37.0	CH
	5	3.98, 1H, m	53.0	CH_2_	3.98, 1H, m	53.0	CH_2_
		3.31, 1H, m			3.27, 1H, m		
	6	5.26, 1H, dd (9.5, 9.5)	127.8	CH	5.26, 1H, dd (9.0, 9.0)	127.9	CH
	7	5.64, 1H, dq (9.5, 7.0)	128.8	CH	5.63, 1H, dq (9.0, 7.0)	128.8	CH
	8	1.68, 1H, d (7.0)	13.5	CH_3_	1.66, 1H, d (7.0)	13.5	CH_3_
Ile	9	–	171.5	C	–	171.5	C
	10	4.64, 1H, m	54.5	CH	4.64, 1H, m1q	54.9	CH
	10-NH	7.31, 1H, brs	–		7.23, 1H, brs	–	
	11	1.88, 1H, m	38.6	CH	1.90, 1H, m	38.2	CH
	12	1.54, 1H, m	25.0	CH_2_	1.55, 1H, m	25.0	CH_2_
		1.29, 1H, m			1.30, 1H, m		
	13	0.90, 3H, d (7.0)	10.8	CH_3_	0.89, 3H, d (7.0)	10.8	CH_3_
	14	1.04, 3H, d (7.0)	15.2	CH_3_	1.02, 3H, d (7.0)	15.3	CH_3_
(βMe)Phe/Phe	15	–	167.9	C	–	171.3	C
	16	4.33, 1H, m	60.8	CH	4.58, 1H, m	55.2	CH
	16-NH	6.85, 1H, brs	–		6.91, 1H, brs	–	
	17	3.02, 1H, m	44.6	CH	2.91, 1H, m	38.0	CH_2_
	17-Me	1.29, 3H, d (6.5)	18.0	CH_3_	2.97, 1H, m		
	18	–	141.9	C	–	136.5	C
	19	7.11, 1H, d (8.5)	129.0	CH	7.11, 1H, d (8.0)	129.5	CH
	20	7.13, 1H, t (8.5)	127.5	CH	7.15, 1H, t (8.0)	129.0	CH
	21	7.03, 1H, t (8.5)	127.6	CH	7.09, 1H, t (8.0)	127.7	CH
	22	7.13, 1H, t (8.5)	127.5	CH	7.15, 1H, t (8.0)	129.0	CH
	23	7.11, 1H, d (8.5)	129.0	CH	7.11, 1H, d (8.0)	129.5	CH
(3-Ncp)Ala I	24	–	169.3	C	–	168.2	C
	25	3.52, 1H, m	52.2	CH	3.55, 1H, m	52.8	CH
	25-NH	6.26, 1H, brs	–		6.37, 1H, brs	–	
	26	0.56, 1H, m	33.4	CH_2_	0.78, 1H, m	33.0	CH_2_
		−0.26, 1H, m			0.13, 1H, m		
	27	0.28, 1H, m	20.2	CH	0.58, 1H, m	20.4	CH
	28	2.92, 1H, m	58.7	CH	3.04, 1H, m	58.6	CH
	29	−0.66, 1H, m	17.7	CH_2_	−0.34, 1H, m	17.4	CH_2_
		1.02, 1H, m			1.13, 1H, m		
Phe/(βMe)Phe	30	–	168.8	C	–	168.3	C
	31	4.48, 1H, m	56.4	CH	4.48, 1H, m	60.6	CH
	31-NH	6.79, 1H, brs	–		6.79, 1H, brs	–	
	32	2.79, 1H, m	38.7	CH_2_	3.67, 1H, m	37.1	CH
		3.38, 1H, m			1.40, 3H, d (6.5)	13.7	CH_3_
	33	–	137.3	C	–	142.6	C
	34	7.22, 1H, m	129.3	CH	7.24, 1H, m	127.9	CH
	35	7.23, 1H, m	127.8	CH	7.25, 1H, m	128.8	CH
	36	7.15, 1H, t (8.5)	127.2	CH	7.17, 1H, t (8.5)	127.3	CH
	37	7.23, 1H, m	127.8	CH	7.25, 1H, m	128.8	CH
	38	7.22, 1H, m	129.3	CH	7.24, 1H, m	127.9	CH
Thr	39	–	171.1	C	–	171.1	C
	40	4.55, 1H, m	58.4	CH	4.52, 1H, m	55.8	CH
	40-NH	8.98, 1H, brs	–		8.95, 1H, brs	–	
	41	5.40, 1H, m	69.4	CH	5.38, 1H, m	69.6	CH
	42	1.52, 3H, m	17.2	CH_3_	1.48, 3H, m	17.3	CH_3_
(3-Ncp)Ala II	43	–	172.2		–	172.1	
	44	5.14, 1H, m	51.2	CH	5.09, 1H, m	51.5	CH
	44-NH	8.16, 1H, brs	–		7.96, 1H, brs	–	
	45	1.80, 1H, m	35.5	CH_2_	1.83, 1H, m	35.2	CH_2_
		1.60, 1H, m			1.61, 1H, m		
	46	1.93, 1H, m	21.7	CH	1.91, 1H, m	21.7	CH
	47	4.04, 1H, m	59.8	CH	4.05, 1H, m	59.6	CH
	48	1.95, 1H, m	17.8	CH_2_	1.95, 1H, m	17.9	CH_2_
		1.02, 1H, m			1.01, 1H, m		
Chpca	49	–	159.7	C	–	160.1	C
	50	–	119.8	C	–	119.8	C
	51	6.68, 1H, d (4.5)	109.8	CH	6.81, 1H, d (4.5)	109.8	CH
	52	6.08, 1H, d (4.5)	103.5	CH	6.13, 1H, d (4.5)	104.0	CH
	53	–	121.5	C	–	120.5	C
	NOH	10.8, 1H, brs	–		10.8, 1H, brs	–	

### 2.2. Bioactivities of the Hormaomycins

Hormaomycin was previously reported to exhibit remarkable antibacterial effects [18]. Therefore, the biological activities of the hormaomycins found here were evaluated with regard to their antimicrobial activities against various pathogenic bacterial strains, including *Staphylococcus aureus* ATCC 25923, *Bacillus subtilis* ATCC 6633, *Kocuria rhizophila* NBRC 12708, *Streptococcus pyogenes* ATCC 19615, *Klebsiella pneumoniae* ATCC10031, *Salmonella enterica* ATCC 14028, *Proteus hauseri* NBRC 3851 and *Escherichia coli* ATCC 25922 (Table 2). Hormaomycin exhibited more potent antibacterial activities against the tested Gram-positive bacteria than against the tested Gram-negative bacteria, particularly against *S. aureus* and *K. rhizophila*, exhibiting MIC values of 0.4 μM and 0.03 μM, respectively. Hormaomycin also exhibited significant antibacterial effects against Gram-negative *P*. *hauseri*. Hormaomycins B and C (**1** and **2**) displayed potent activity against *K. rhizophila*, but showed antibacterial activities against the tested bacteria that were generally 4–32-times weaker than those of hormaomycin. Accordingly, it was hypothesized that the existence of the methyl groups at C-17 and C-32, which form (βMe)Phe, play an important role in the antibacterial potency of the hormaomycins. Further investigations of the antifungal activity were conducted against the pathogenic fungi *Aspergillus fumigatus*, *Trichophyton rubrum*, *T*. *mentagrophytes* and *Candida albicans*, but no significant inhibitory activity was observed to result from the hormaomycin treatments (MIC > 100 μM).

**Table 2:** Antibacterial activity data of the hormaomycins.

Compound	MICs (μM)							
	Gram-Positive				Gram-Negative			
	*S. aureus*	*B. subtilis*	*K. rhizophila*	*S. pyogenes*	*K. pneumoniae*	*S. enterica*	*P. hauseri*	*E. coli*
Hormaomycin	0.4	1.8	0.03	1.8	>113	>113	0.9	>113
Hormaomycin B	7	14	0.4	14	>115	29	29	>115
Hormaomycin C	7	56	0.23	8	>114	114	14	>114
Ampicillin	<0.17	0.17	<0.17	0.17	45.9	3.7	<0.17	11.5

## 3. Experimental Section

### 3.1. General Experimental Procedures

Optical rotations were measured using a JASCO P-200 polarimeter with a 1-cm cell. IR spectra were obtained using a Thermo NICOLET iS10 spectrometer (sodium light source, JASCO, Easton, PA, USA). UV spectra were acquired using a Perkin Elmer Lambda 35 UV/VIS spectrometer (Perkin Elmer, Waltham, MA, USA). CD spectra were collected with an Applied Photophysics Chirascan-Plus (Applied Photophysics, Leatherhead Surrey, UK) with a 2-mm cell. Electrospray ionization (ESI) low-resolution LC/MS data were recorded on an Agilent Technologies 6130 Quadrupole mass spectrometer connected to an Agilent Technologies 1200 series high-performance liquid chromatography (HPLC) instrument (Agilent Technologies, Santa Clara, CA, USA). High-resolution fast atom bombardment (HR-FAB) mass spectra were recorded using a JEOL JMS-600W high-resolution mass spectrometer (Jeol, München, Germany) at the National Center for Inter-university Research Facilities at Seoul National University (NCIRF). ^1^H, ^13^C and 2D NMR spectra (See Supplementary Information Figures S1–S18) were recorded on Bruker Avance 600 MHz spectrometers (Bruker, Billerica, MA, USA) at the NCIRF.

### 3.2. Isolation, Cultivation and Extraction of Bacteria

A sediment sample was collected from the Mohang mudflat in Buan, Korea. The sample was dried at room temperature for 3 h. The dry sediment (1 g) was diluted in 4 mL of sterilized artificial seawater. The mixture was spread on actinomycete isolation agar, A4 medium (1 L of seawater, 18 g of agar and 100 mg/L cycloheximide) and A5 medium (750 mL of seawater, 250 mL of distilled water, 18 g of agar and 100 mg/L cycloheximide) by stamping and spreading. The single strain SNM55 was isolated on A5 medium. The strain SNM55 (GenBank Accession No. KP133063) was phylogenetically identified as a *Streptomyces* sp. (most closely related to *Streptomyces javenis* by 99% identity) based on the 16S rDNA sequence analysis. The bacterium was incubated in 50 mL of YEME medium (4 g of yeast extract, 10 g of malt extract and 4 g of glucose in 1 L of artificial seawater) in a 125-mL Erlenmeyer flask for 3 days on a rotary shaker at 160 rpm and 30 °C. Then, 10 mL of the culture were inoculated into 200 mL of YEME medium in a 500-mL Erlenmeyer flask. The strain SNM55 was further cultivated for 2 days, and 20 mL of the liquid culture were transferred to 1 L of YEME liquid medium in a 2.8-L Fernbach flask (12 ea × 1 L, total volume of 12 L). After incubating the bacterial culture for 10 days, the entire culture (12 L) was extracted with 16 L of ethyl acetate. The ethyl acetate layer was collected, and residual water in the organic layer was removed by adding anhydrous sodium sulfate. The extract was concentrated *in vacuo*. The entire procedure was repeated 6 times (72 L culture in total) to yield 4 g of dry material for chemical study and bioassays.

### 3.3. Isolation of the Hormaomycins

The extract of SNM55 (one sixth of the 4 g of dry material collected) was absorbed on Celite and loaded on a 2g Sep-Pak C_18_ cartridge. Then, the extract was fractionated with 20 mL each of 20%, 40%, 60%, 80% and 100% MeOH in water and 1:1 MeOH/dichloromethane. The hormaomycins were detected in the 80% and 100% MeOH/water fractions by LC/MS. The 80% and 100% fractions were combined into one vial and subjected to semi-preparative reversed-phase HPLC (Kromasil C_18_ (2): 250 × 10 mm, 5 μm) using a gradient solvent system (65% MeOH/H_2_O to 90% MeOH/H_2_O over 40 min and 100% MeOH from 40 min to 50 min, UV 280 nm detection, flow rate: 2mL/min). Three fractions containing the hormaomycins were collected at retention times of 43 min, 45 min and 52 min. Each of the hormaomycins was purified by an isocratic solvent system (75% acetonitrile/H_2_O, UV 280 nm detection, flow rate: 2mL/min) using a reversed-phase C_18_ column (Kromasil C_18_ (2): 250 × 10 mm, 5 μm). Hormaomycin B (**1**) (4 mg), hormaomycin C (**2**) (3 mg) and hormaomycin (6 mg) eluted as pure compounds at retention times of 14.5 min, 15.2 min and 18.4 min, respectively, under the final purification conditions.

Hormaomycin B (**1**): [α]_D_ 21.9, (c 0.025, MeOH); UV (MeOH) λ_max_ (log ε) 208 (4.57), 271 (4.08) nm; IR (neat) ν_max_ 3309, 2958, 1626, 1543, 1369 cm^−1^; for ^1^H and ^13^C NMR data, see Table 1; HRFABMS *m*/*z* 1115.4611 [M + H]^+^ (calcd. for C_54_H_68_ClN_10_O_14_ 1115.4605).

Hormaomycin C (**2**): [α]_D_ 29.9, (c 0.025, MeOH); UV (MeOH) λ_max_ (log ε) 208 (4.57), 271 (4.09) nm; IR (neat) ν_max_ 3343, 2931, 1627, 1554, 1362 cm^−1^; for ^1^H and ^13^C NMR data, see Table 1; HRFABMS *m*/*z* 1137.4432 [M + Na]^+^ (calcd. for C_54_H_67_ClN_10_O_14_Na 1137.4424).

### 3.4. Antibacterial Activity Assay

Gram-positive bacteria (*S. aureus* ATCC 25923, *B. subtilis* ATCC 6633, *S. pyogenes* ATCC 19615 and *K. rhizophila* NBRC 12708) and Gram-negative bacteria (*K. pneumoniae* ATCC10031, *S. enterica* ATCC 14028, *E. coli* ATCC 25922 and *P. hauseri* NBRC 3851) were used for antimicrobial activity tests. Bacteria were grown overnight in Mueller Hinton (MH) broth at 37 °C, harvested by centrifugation and washed twice with sterile distilled water. Stock solutions of the hormaomycins were prepared in DMSO. Each stock solution was diluted with MH broth (5% lysed sheep blood for *S. pyogenes*) to give serial 2-fold dilutions in the range of 128 to 0.06 μg/mL. Aliquots (10 μL) of the broth containing approximately 5 ×10^5^ colony-forming units (cfu)/mL of the bacteria were added to each well of a 96-well microtiter plate. The plates were incubated for 24 h at 37 °C. The minimum inhibitory concentration (MIC) values were determined as the lowest concentration of test compound that inhibited bacterial growth. Ampicillin was used as a reference compound.

### 3.5. Antifungal Activity Assay

Potato dextrose agar (PDA) was used to cultivate *C. albicans* ATCC 10231. After incubation for 48 h at 28 °C, yeast cells were harvested by centrifugation and washed twice with sterile distilled water. *A. fumigatus* HIC 6094, *T. rubrum* NBRC 9185 and *T. mentagrophytes* IFM 40996 were plated on PDA and incubated for 2 weeks at 28 °C. Spores were harvested and washed twice with sterile distilled water. Fungal cells were resuspended in RPMI 1640 broth (Difco) to obtain an initial inoculum size of 10^5^ spores/mL. In each well of a 96-well plate, 90 μL of cells (10^4^ cells/mL) were mixed with the test compound solutions (hormaomycin and hormaomycins B and C) in 5% DMSO. A culture with DMSO (0.5%) was used as a solvent control, and a culture supplemented with amphotericin B was used as a positive control.

## 4. Conclusions

As one approach to maximize microbial chemical diversity, altering the culture conditions of a chemically-prolific marine actinomycete strain (SNM55) led us to discover hormaomycins B and C (**1** and **2**). These hormaomycins are structurally-unique cyclic depsipeptides that incorporate various unusual units, such as 4-(*Z*)-propenylproline, 3-(2-nitrocyclopropyl)alanine, β-methylphenylalanine and 5-chloro-1-hydroxypyrrol-2-carboxylic acid. The hormaomycins inhibited various pathogenic Gram-positive and Gram-negative bacteria. To the best of our knowledge, hormaomycins B and C are the first natural analogues of hormaomycin. The discovery of these new members of the hormaomycin family provides additional evidence that marine actinomycetes possess the potential to produce still-untapped bioactive secondary metabolites, which can be synthesized through diverse biosynthetic pathways under appropriate culture conditions.

## Supplementary Files

Supplementary File 1
