# Supplementary material for: Hormaomycins B and C: New Antibiotic Cyclic Depsipeptides from a Marine Mudflat-Derived Streptomyces sp
Source: Mar Drugs. 2015 Aug 14;13(8):5187–200. doi: 10.3390/md13085187 (PMC4557019; doi:10.3390/md13085187)
Supplement: Supplementary File 1 [file marinedrugs-13-05187-s001.docx]

**Supplementary Information**


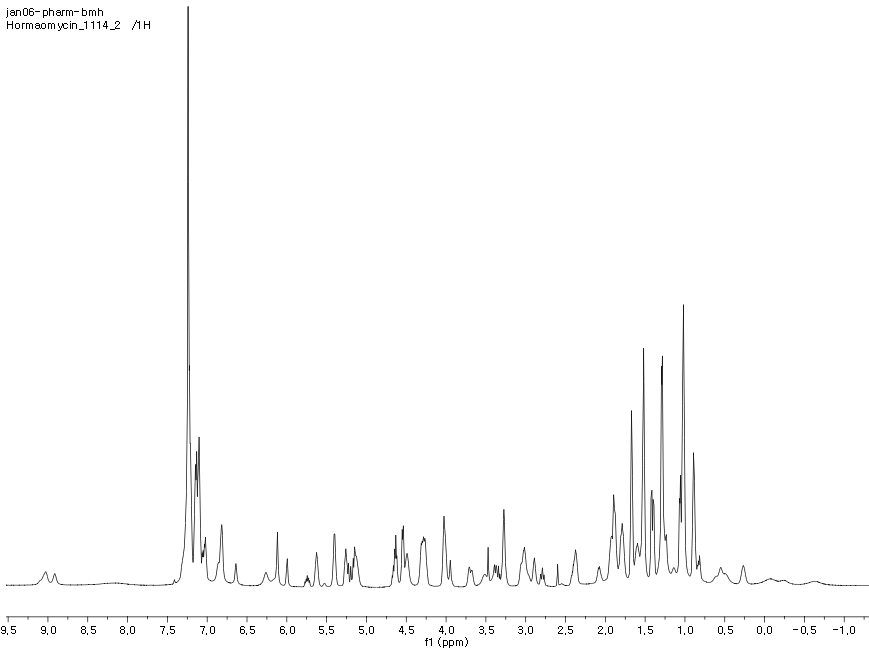


**Figure S1.** ^1^H NMR spectrum (600 MHz) of hormaomycin B (**1**) in CDCl_3_.


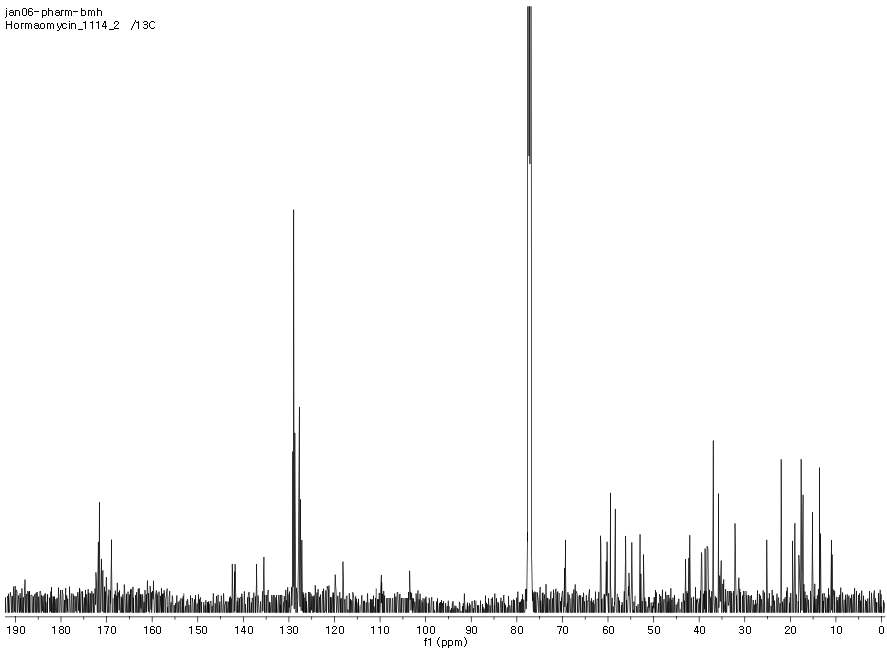


**Figure S2.** ^13^C NMR spectrum (150 MHz) of hormaomycin B (**1**) in CDCl_3_.


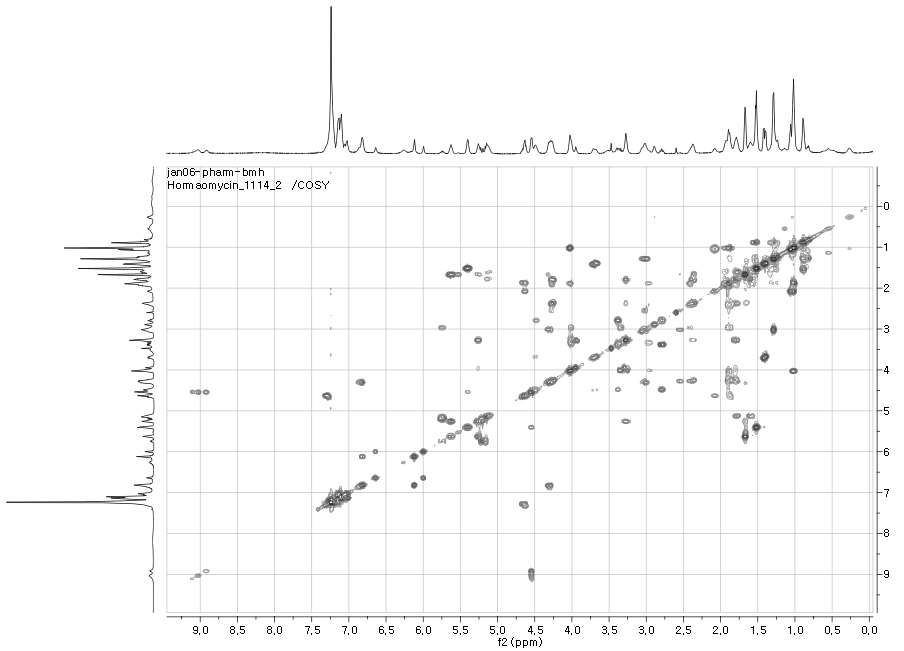


**Figure S3.** COSY spectrum (600 MHz) of hormaomycin B (**1**) in CDCl_3_.


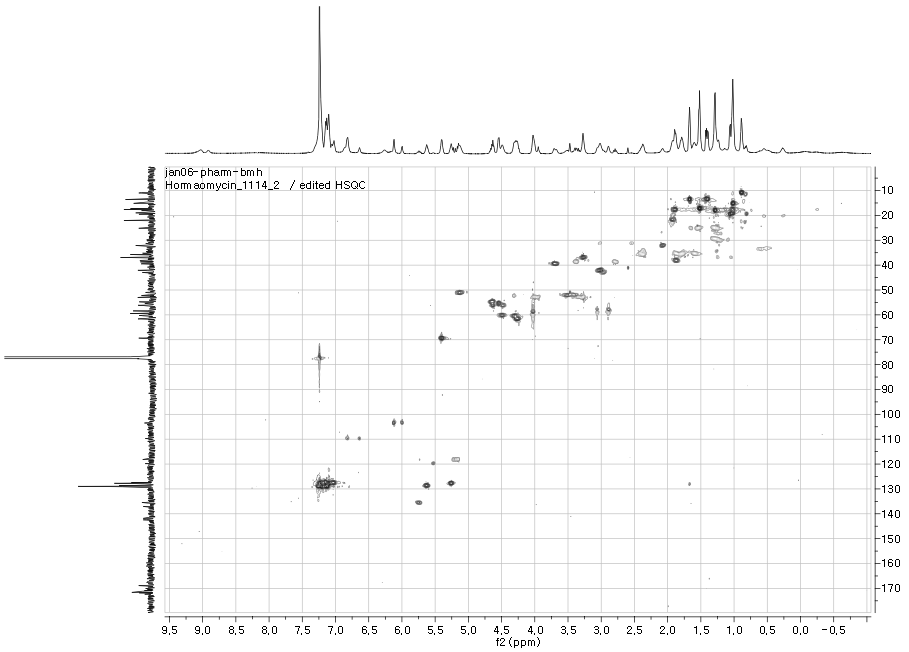


**Figure S4.** HSQC spectrum (600 MHz) of hormaomycin B (**1**) in CDCl_3_.


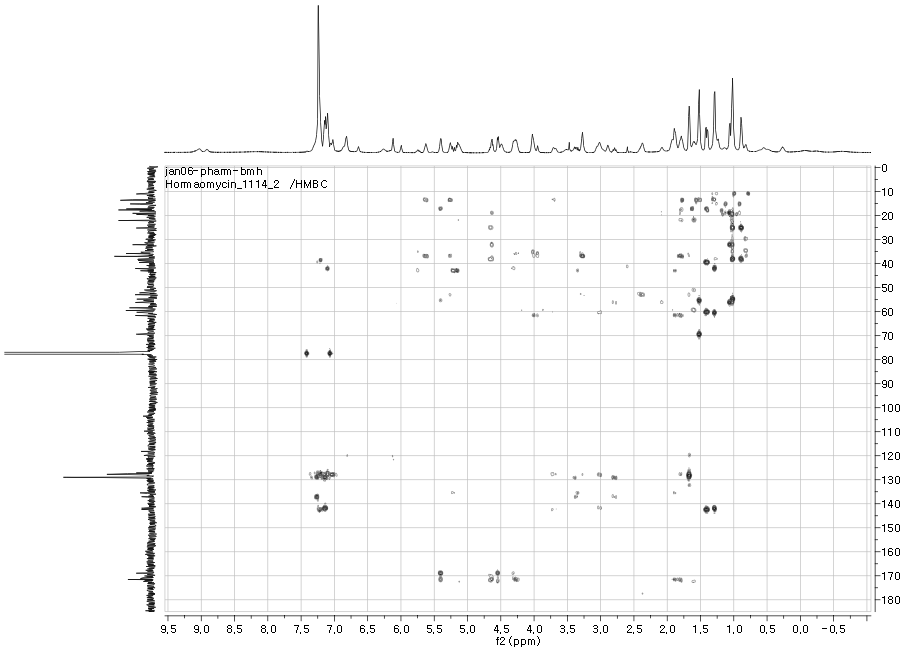


**Figure S5.** HMBC spectrum (600 MHz) of hormaomycin B (**1**) in CDCl_3_.


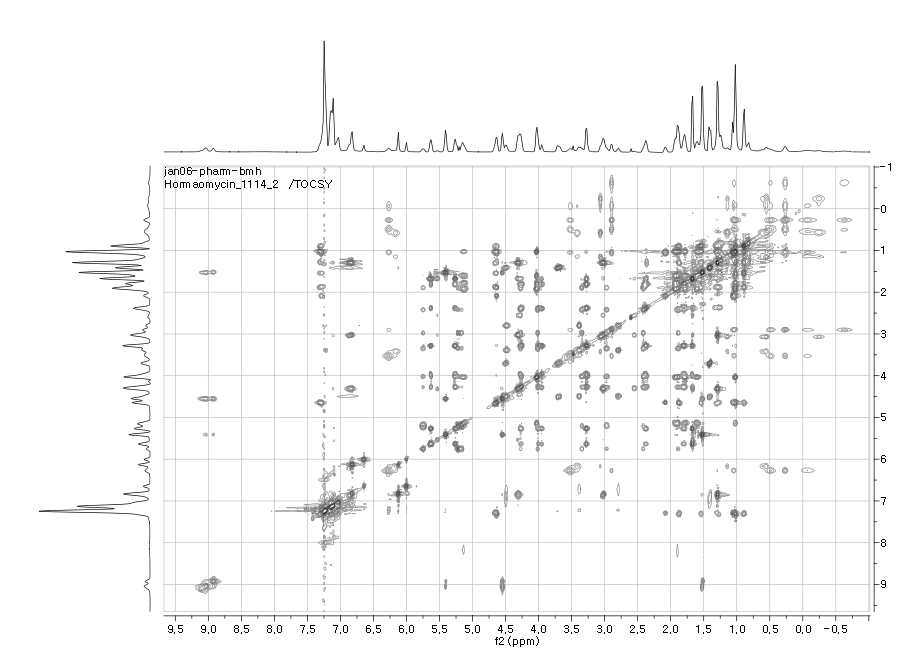


**Figure S6.** TOCSY spectrum (600 MHz) of hormaomycin B (**1**) in CDCl_3_.


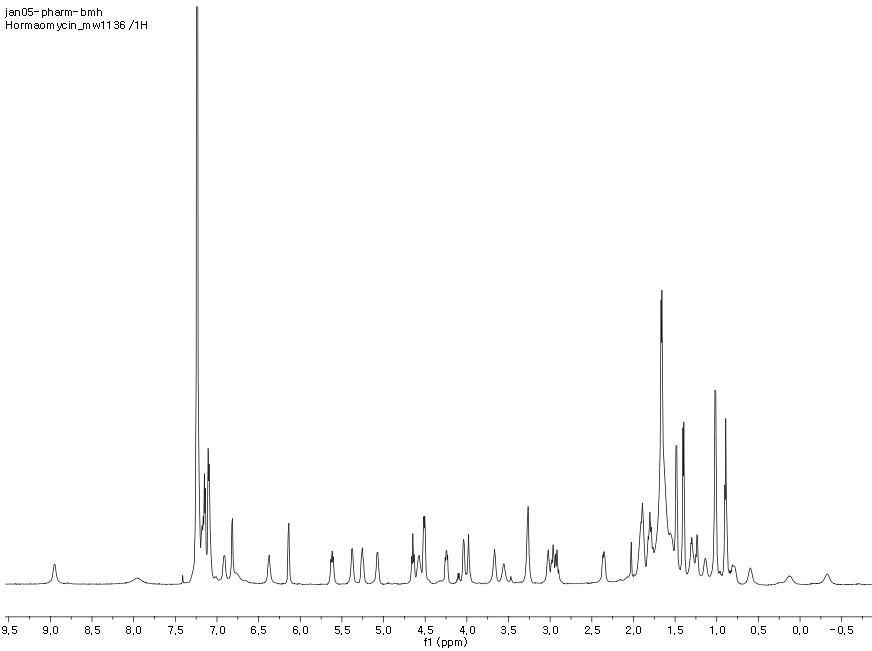


**Figure S7.** ^1^H NMR spectrum (600 MHz) of hormaomycin C (**2**) in CDCl_3_.


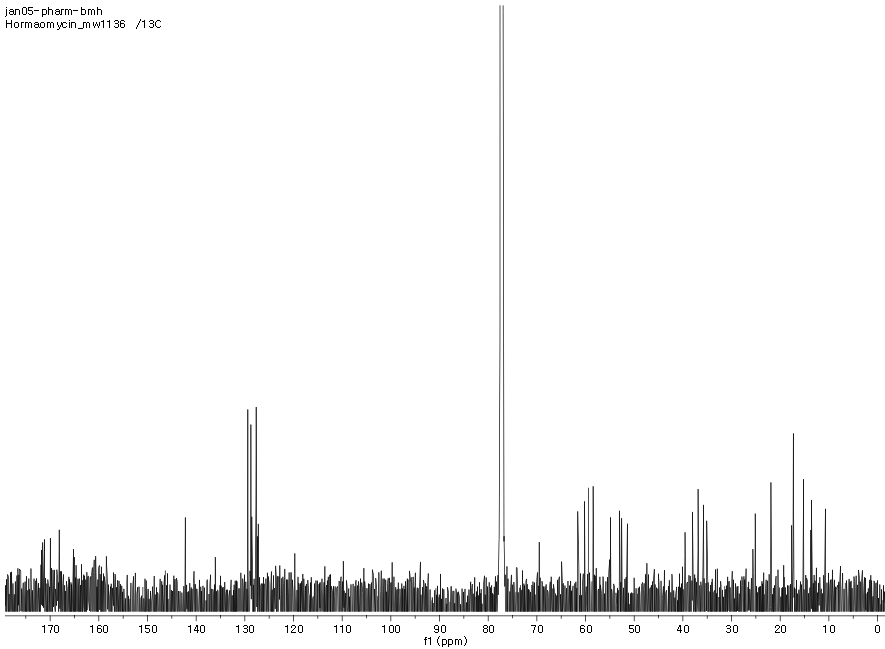


**Figure S8.** ^13^C NMR spectrum (150 MHz) of hormaomycin C (**2**) in CDCl_3_.


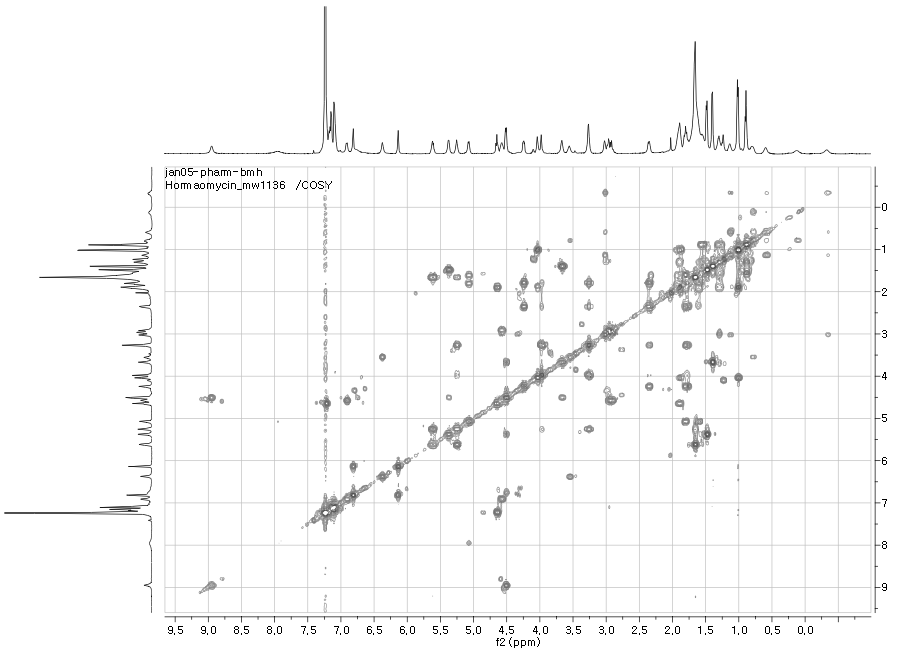


**Figure S9.** COSY spectrum (600 MHz) of hormaomycin C (**2**) in CDCl_3_.


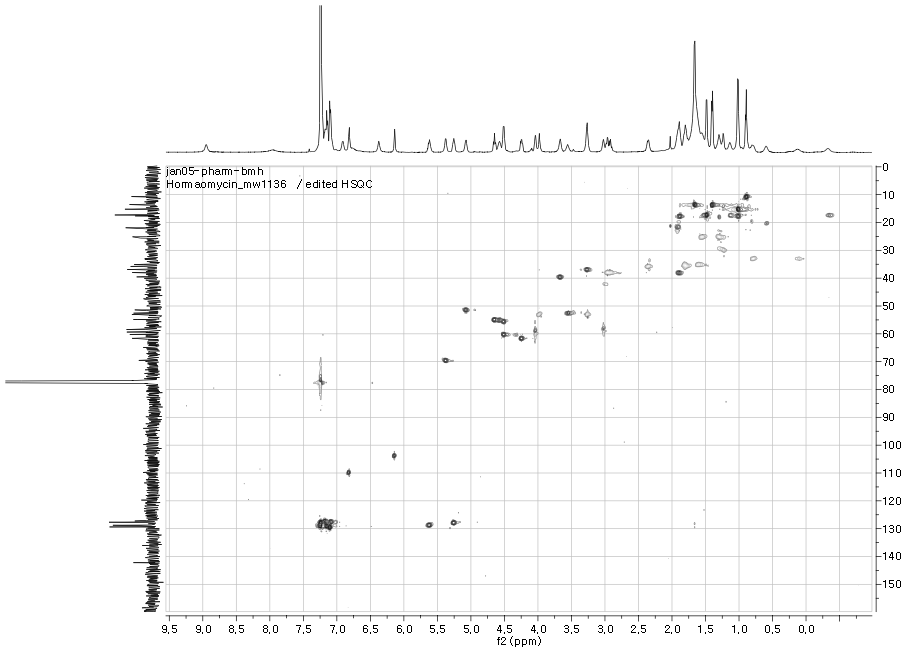


**Figure S10.** HSQC spectrum (600 MHz) of hormaomycin C (**2**) in CDCl_3_.


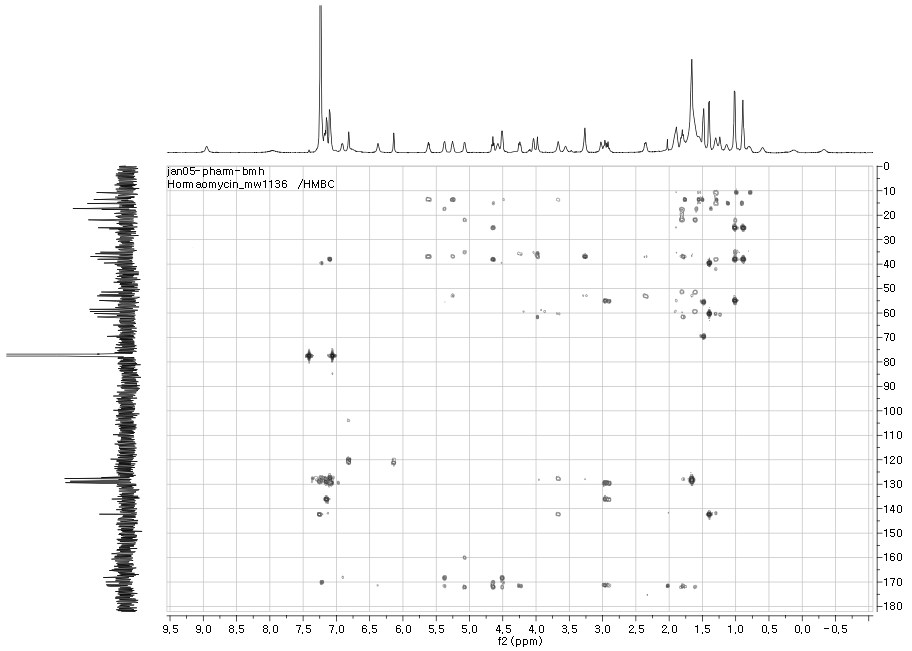


**Figure S11.** HMBC spectrum (600 MHz) of hormaomycin C (**2**) in CDCl_3_.


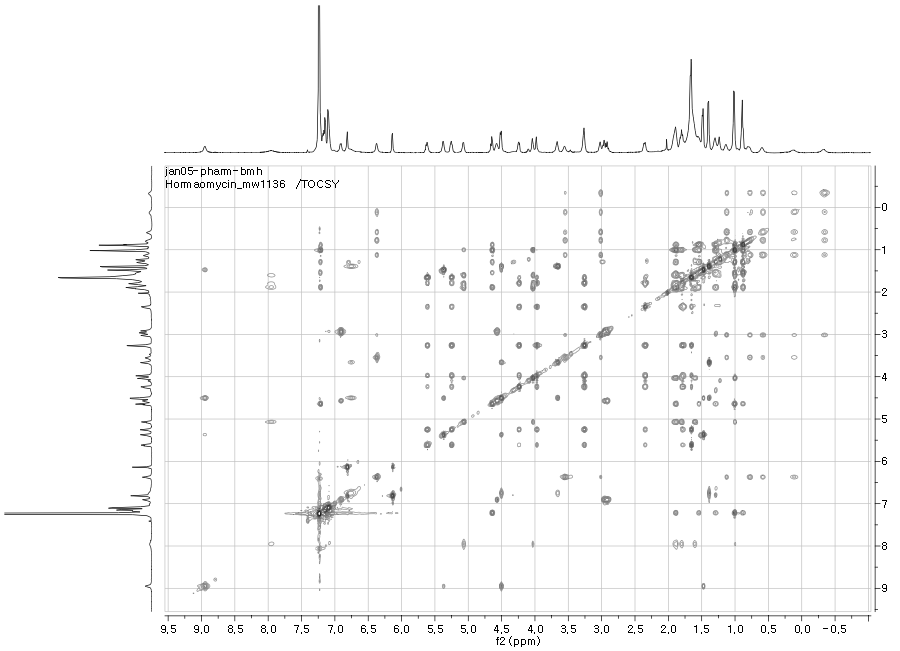


**Figure S12.** TOCSY spectrum (600 MHz) of hormaomycin C (**2**) in CDCl_3_.


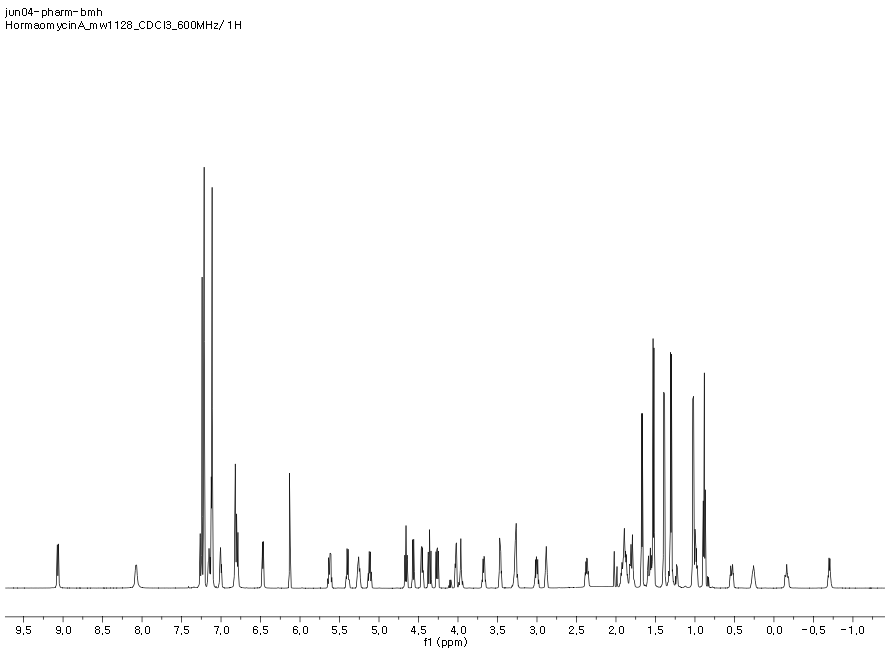


**Figure S13.** ^1^H NMR spectrum (600 MHz) of hormaomycin in CDCl_3_.


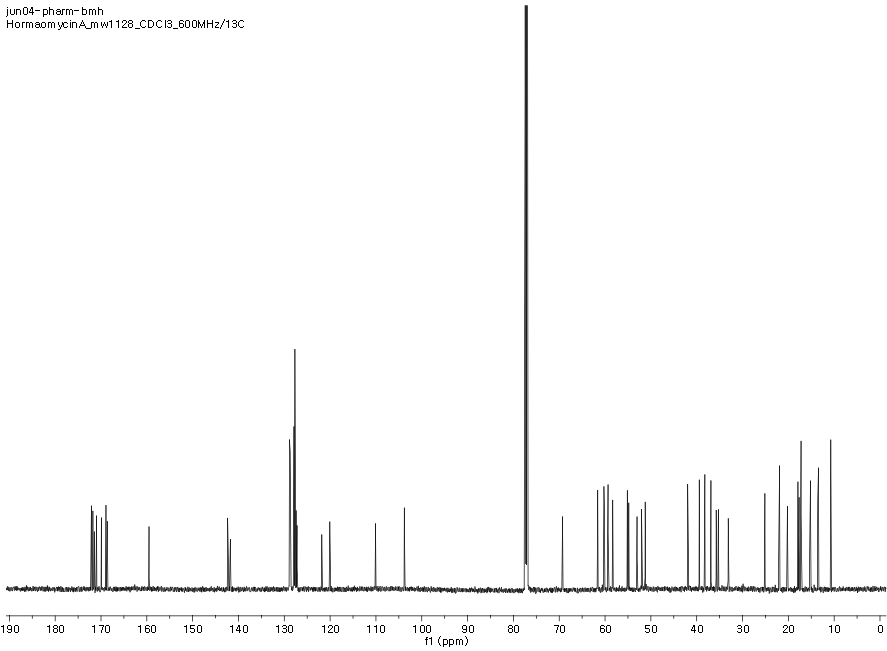


**Figure S14.** ^13^C NMR spectrum (150 MHz) of hormaomycin in CDCl_3_.


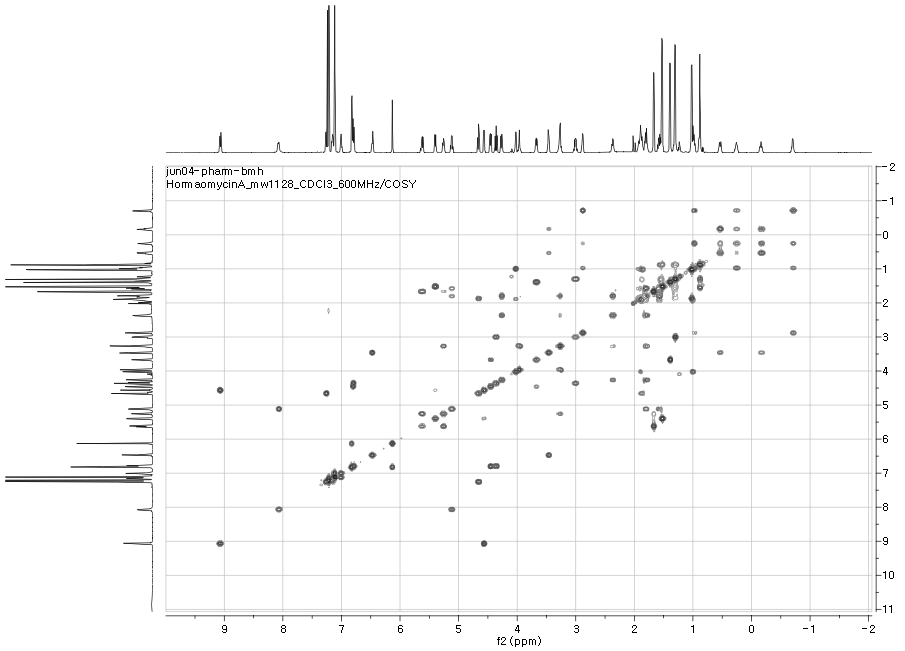


**Figure S15.** COSY spectrum (600 MHz) of hormaomycin in CDCl_3_.


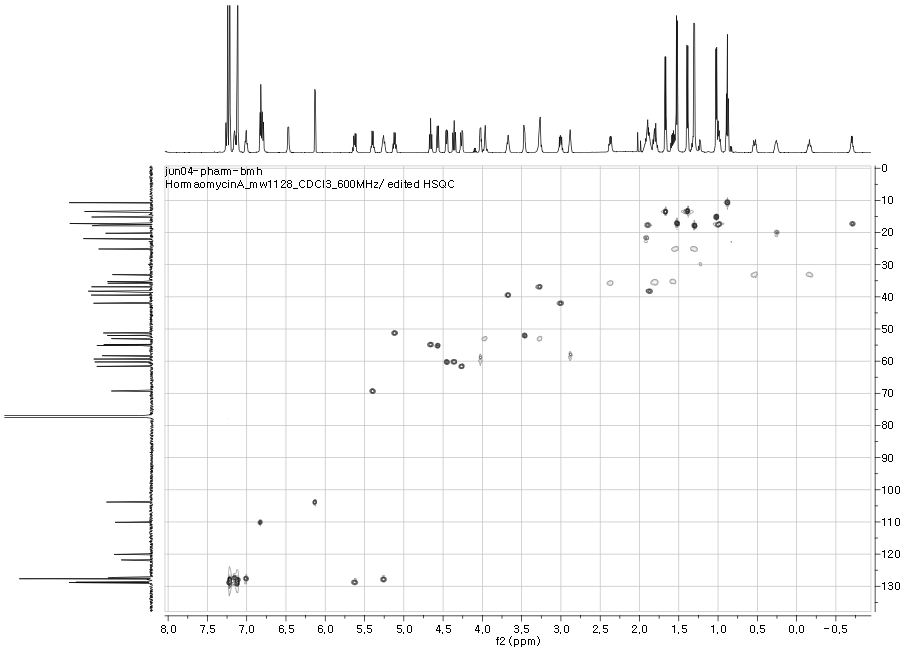


**Figure S16.** HSQC spectrum (600 MHz) of hormaomycin in CDCl_3_.


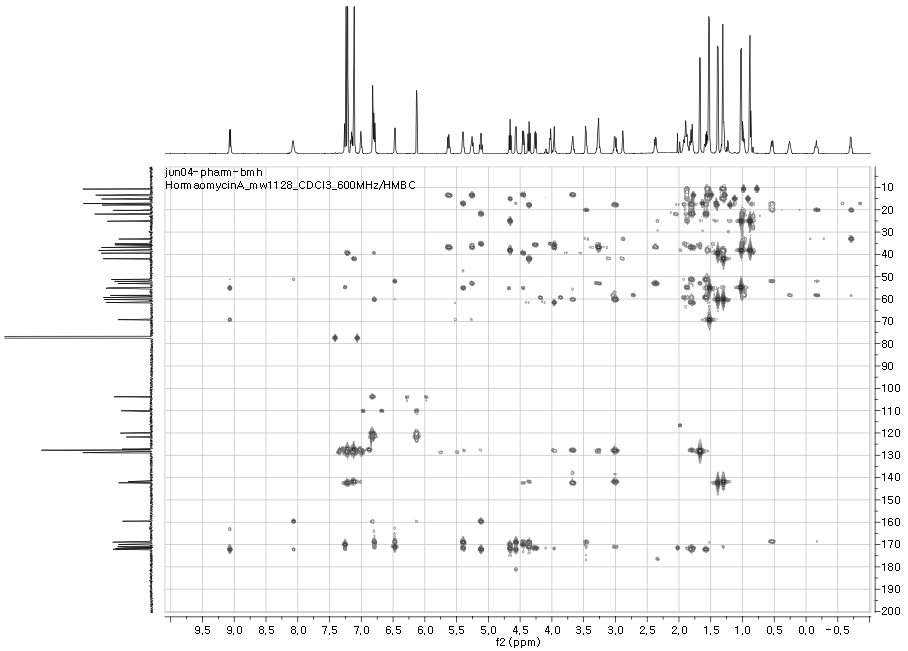


**Figure S17.** HMBC spectrum (600 MHz) of hormaomycin in CDCl_3_.


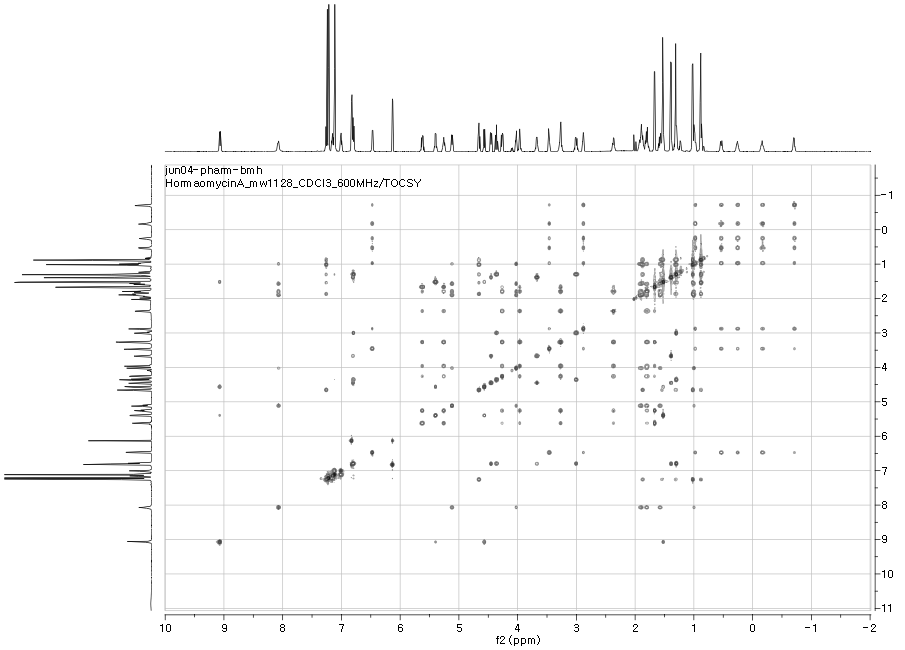


**Figure S18.** TOCSY spectrum (600 MHz) of hormaomycin in CDCl_3_.
